# Strategies to improve response rates to patient reported outcome measures in a surgical RCT

**DOI:** 10.1186/1745-6215-16-S2-P123

**Published:** 2015-11-16

**Authors:** Jessica Wood, Hanne Bruhn, Jonathan A Cook, Alison McDonald, John Norrie, Angus J M Watson

**Affiliations:** 1 University of Aberdeen, Aberdeen, UK; 2 University of Oxford, Oxford, UK; 3 NHS Highland, Inverness, UK

## 

Difficulties achieving high response rates to patient reported outcome measures (PROMs) within clinical trials are well recognised. The NIHR HTA funded eTHoS trial, which compares Stapled Haemorrhoidopexy with Traditional Haemorrhoidectomy for the treatment of grade II-IV haemorrhoids, is no exception.

Early response rates to postal PROMs on quality of life (distributed at 12 and 24 months post-randomisation) showed that their return was only 67% (112/166). Over the period of the study various methods have been employed to improve response rates including monetary incentives. The current literature base suggests that that this may improve such responses. High value incentives may have a greater overall effect than lower value incentives, however evidence from RCTs is limited. We examined whether introducing monetary incentives affected response rates in a subset of the eTHoS cohort.

Two monetary incentive studies were conducted. In the first, participants (n=326) were randomly allocated to one of 4 groups: 1) no incentive; 2) £5 gift voucher enclosed with 12 and 24 month questionnaires; 3) £5 gift voucher at 12 months only; 4) £5 gift voucher at 24 months only.

In the second (ongoing), participants are sent a £30 gift voucher on receipt of completed questionnaires (12 and 24 months).

We will present the results from both studies. In addition, other methods adopted by the trial team, such as a shortened reminder questionnaire, enabling participants to complete questionnaires online, and administering a study newsletter one week before the 12 and 24 month questionnaires were sent will be discussed.

